# Ultrasound-Assisted Green Synthesis of Dialkyl Peroxides under Phase-Transfer Catalysis Conditions

**DOI:** 10.3390/molecules25010118

**Published:** 2019-12-28

**Authors:** Daniel Kopeć, Stefan Baj, Agnieszka Siewniak

**Affiliations:** Department of Chemical Organic Technology and Petrochemistry, Silesian University of Technology, Krzywoustego 4, 44-100 Gliwice, Poland

**Keywords:** dialkyl peroxide, ultrasound irradiation, phase-transfer catalysis, tri-liquid PTC system, third-liquid phase

## Abstract

The dialkyl peroxides, which contain a thermally unstable oxygen–oxygen bond, are an important source of radical initiators and cross-linking agents. New efficient and green methods for their synthesis are still being sought. Herein, ultrasound-assisted synthesis of dialkyl peroxides from alkyl hydroperoxides and alkyl bromides in the presence of an aqueous solution of an inorganic base was systematically studied under phase-transfer catalysis (PTC) conditions. The process run in a tri-liquid system in which polyethylene glycol as a phase-transfer catalyst formed a third liquid phase between the organic and inorganic phases. The use of ultrasound provided high yields of organic peroxides (70–99%) in significantly shorter reaction times (1.5 h) in comparison to reaction with magnetic stirring (5.0 h). In turn, conducting the reaction in the tri-liquid PTC system allowed easy separation of the catalyst and its multiple use without significant loss of activity.

## 1. Introduction

The phase-transfer catalysis (PTC) is a well-established technique in the synthesis of compounds with important practical applications in pharmaceuticals, agrochemicals, fragrances, dyes, monomers, and others [1]. It is a special type of heterogeneous catalysis in which the reaction occurs between reagents that are located in two phases in the presence of a phase-transfer catalyst. The role of the catalyst is to transport one of the reagents in the active state from a usually inorganic phase to an organic phase, where the reaction with the organic reagent takes place [2]. PTC catalysts are often quaternary ammonium salts, crown ethers, or polyethylene glycols. The benefits of using PTC are minimization or elimination of organic solvents, the use of easily accessible, cheap and often safer reagents (e.g., NaOH, KOH, K_2_CO_3_, etc. instead of NaH, t-BuOK, etc.), high yield and high purity of products obtained, and simple synthesis procedure, to name a few.

The dialkyl peroxides are readily used as radical polymerization initiators, e.g., in the polymerization of olefins and as crosslinking agents, e.g., for ethylene–propylene copolymers, silicone rubbers, and others [3,4].

A convenient way to obtain unsymmetrical dialkyl peroxides is the reaction between alkyl hydroperoxides and alkyl halides in the presence of inorganic bases, e.g., KOH and NaOH. This reaction proceeds in a two-phase system. Maillard et al. carried out the reaction with a solid potassium hydroxide, using polyethylene glycol [5] or benzyltriethylammonium chloride [6] as the phase-transfer catalyst. These methods allowed them to obtain peroxide yields ranging from 18% to 80% after reaction times from 0.5 to 72 h. The problem that occurs with the use of soluble PTC catalysts concerns the difficulties of separating the catalyst from the reaction mixture. It may affect product purity and process costs.

In previous work, we described the efficient method of the dialkyl peroxides synthesis from alkyl hydroperoxides and alkyl bromides by using the immobilized PTC catalysts [7]. This enabled easy separation and reuse of the catalyst. However, to avoid the additional costs associated with the immobilization of the catalyst on a solid support, we applied a tri-liquid PTC technique to obtain dialkyl peroxides [8,9]. In this technique the liquid PTC catalyst is predominately located in a third liquid phase and can be easily separated from the reaction mixture [10,11,12].

In the continuation of our research interests in the development of green synthesis of dialkyl peroxide, we used ultrasound to accelerate the process of their synthesis in the tri-liquid PTC system using nontoxic, cheap, and biodegradable polyethylene glycols as the PTC catalysts. In the literature, there are many examples which prove that the combination of PTC and ultrasound contributes to the intensification of the process [13,14,15,16,17,18,19].

## 2. Results and Discussion

**Scheme 1:**
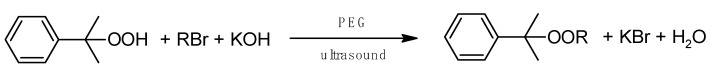
A model reaction.

Research on the influence of ultrasound on the course of the synthesis of dialkyl peroxides was carried out on the example of the reaction between 1-bromobutane and cumyl hydroperoxide (CHP) (Scheme 1). Polyethylene glycols and their derivatives were used as phase-transfer catalysts. The reaction was carried out in such a way that the catalyst formed a third liquid phase. The other two liquid phases were CHP dissolved in cyclohexane and a concentrated inorganic base solution.

### 2.1. Effect of Ultrasound

We started our research by comparing the influence of two techniques on the course of the model reaction: an ultrasound irradiation and a magnetic stirring. In the case of the first technique, the process was carried out in a thermostated ultrasound bath (45 kHz, 350 W), while, in the second one, it was carried out with a magnetic stirrer (500 rpm). We chose the speed of 500 rpm based on our previous research, which showed that above 400 rpm the rate of the model reaction in the tri-liquid system was independent of the stirring speed. The other reaction conditions were the same (50 °C; 50% KOH; PEG MM 2000—polyethylene glycol monomethyl ether CH_3_O(CH_2_CH_2_O)_44_CH_3_; cyclohexane as an organic solvent).

The results presented in Table 1 clearly show that the use of ultrasound significantly caused a decrease in the required reaction time. A similar peroxide yield was achieved after 1.5 h instead of 5.0 h, as it was in the case of using magnetic stirring. This result confirmed that cavitation had a positive effect on the intensification of the process of obtaining dialkyl peroxides in a tri-liquid system where all reagents and catalyst remained in the separate phases.

### 2.2. Effect of Catalyst Structure

PEGs with different chain lengths and different end groups –OH or –CH_3_ were used for the tests. In Table 2 PEG means polyethylene glycol with the formula HO(CH_2_CH_2_O)_n_H, PEG MM means polyethylene glycol monomethyl ether HO(CH_2_CH_2_O)_n_CH_3_, and in turn PEG DM is polyethylene glycol dimethyl ether CH_3_O(CH_2_CH_2_O)_n_CH_3_, whereas the number after the catalyst abbreviation represents the average molecular weight of PEG.

**Scheme 2:**
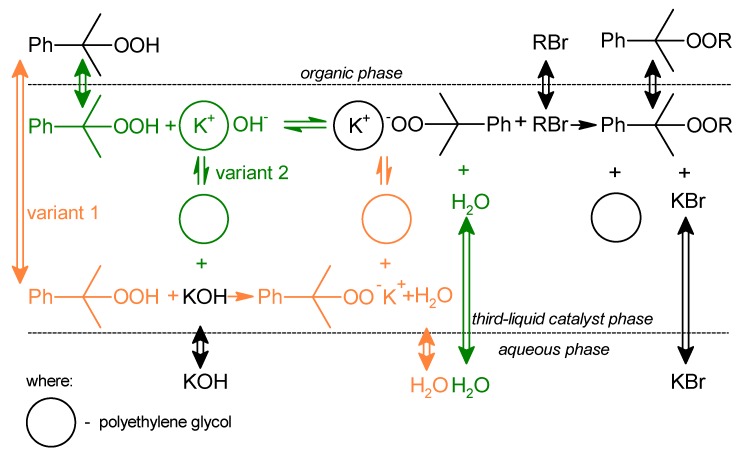
Proposed scheme of the model reaction course.

Based on the results in Table 2, it might be observed that the reaction yield increased with increasing length of the polyethylene oxide chain. The role of the catalyst is a complexation of potassium cation (Scheme 2). Polyethylene glycols with low “n” values are too short to form complex with potassium cation. Catalysts with higher “n” values, e.g., PEG MM 2000, are long enough to easily complex even two cations at once, which results in their increasing activity and in higher peroxide yield [20].

When catalysts with similar molecular weights and chain lengths were compared (entries 2, 5, and 10 in Table 2), it appeared that the catalyst end groups also affected the dialkyl peroxide yield. The most active catalyst in the reaction was that which had both –CH_3_ and –OH as end groups. In further studies, PEG MM 2000 was used as a catalyst. The use of less than 10% of the catalyst relative to CHP resulted in a significant drop in the peroxide yield (entries 7, 8, and 9).

### 2.3. Effect of a Kind, Concentration, and Amount of Inorganic Base and a Kind of Organic Solvent

The comparison of 50% solutions of two bases KOH and NaOH (entries 1 and 2 in Table 3), showed that better peroxide yield was obtained by using a solution of potassium hydroxide. Most likely, this is due to the fact that KOH is a stronger base than NaOH. In addition, PEGs more easily form complexes with potassium cations than sodium ones [1]. Moreover, in the case of using a 50% NaOH solution, the reaction mixture became turbid and viscous, which might hinder mixing of the reaction mixture.

In the case of KOH solutions of varying concentrations, it can be seen that, as the concentration increased, the reaction yield also increased. With a 50% KOH solution the peroxide yield was close to 100%.

The effect of 50% aqueous solution of KOH on the course of the model reaction was examined for molar ratio KOH to CHP of 20:1, 10:1, and 5:1 (entries 2, 5, and 6 in Table 3). The other reaction conditions remained unchanged. The peroxide yield increased with increasing molar ratio of KOH to CHP. It should be emphasized that, in each case, the formation of a three-phase system was observed.

The selection of a suitable solvent is also crucial for the course of the model reaction, as well as for the catalyst to remain in the third liquid phase immiscible with the others. For the tests, solvents of various polarity were used, such as cyclohexane, toluene, and chlorobenzene. Only in the case of cyclohexane, the three-phase system was stable even after the completion of the reaction. If toluene was used, the tri-liquid system disappeared after the reaction and the peroxide yield was low (Table 4). For chlorobenzene, only the two-phase system was observed.

### 2.4. Synthesis of Dialkyl Peroxides and Recycling of Catalyst

We examined the effect of the alkyl chain length of the alkyl bromide on the peroxide yield under the most favorable reaction conditions obtained on the basis of the present study (50% KOH, KOH to CHP of 20:1, PEG MM 2000, and cyclohexane). It can be observed that the peroxide yield decreased with the lengthening of the alkyl chain (Table 5). However, all peroxides were obtained with high yields (70–99%).

To demonstrate the practical potential of the presented method, the recycling studies of the catalysts were also investigated. In the case of the tri-liquid system, separation of the reaction mixture was easy, using just a simple phase separation. The separated catalyst was used for the next process. Only a slight decrease in peroxide yield was observed.

### 2.5. Proposed Scheme of the Model Reaction Course

It is assumed that the reactions carried out in the tri-liquid PTC system occur mainly in the catalyst phase. This requires that all reagents diffuse into the middle phase. In such a system, the model reaction involves several steps: (1) formation of potassium hydroperoxide salt at the interface between the organic and inorganic phases and then the formation of a complex with polyethylene glycol PEG-K^+^PhC(CH_3_)_2_OO^-^ (variant 1), or PEG forms a complex with KOH and then reacts with cumyl hydroperoxide to form a complex PEG-K^+^PhC(CH_3_)_2_OO^-^ (variant 2); (2) diffusion of alkyl bromide to the catalyst phase; (3) reaction between alkyl bromide and PEG-K^+^PhC(CH_3_)_2_OO^–^ (4) diffusion of the product to the organic phase.

## 3. Materials and Methods

### 3.1. Chemicals

All applied organic reagents and solvents were of reagent grade purity and were used without further purification, unless stated otherwise. The 1-Methyl-1-phenylethyl hydroperoxide (cumyl hydroperoxide, CHP) was purified by the method described elsewhere [21].

The 1-Bromobutane (>98%) and cyclohexane were distilled prior to being used for synthesis. PEG (polyethylene glycol) 200, PEG 600, and PEG 1000 were purchased from Alfa Aesar GmbH & Co KG (Karlsruhe, Germany). PEG MM (polyethylene glycol monomethyl ether) 350, PEG MM 550, PEG MM 1100, PEG MM 2000, PEG DM polyethylene glycol dimethyl ether) 500, and PEG DM 1000 were purchased from Fluka Chemie GmbH (Steinheim, Germany).

### 3.2. Apparatus

UPLC was performed on Acquity Waters chromatography equipped with an Acquity Waters PDA detector and an Acquity UPLC BEH C18 column (100 mm × 2.1 mm, 1.7 μm) (Waters Corp., Milford, MA, USA). Reactions were performed in an ultrasonic bath EMAG Emmi 60 (Mörfelden-Walldorf, Germany).

### 3.3. Experimental Procedure

Typical reaction was carried out in a two-neck round bottom flask (10 cm^3^) (Vitromin S.C., Kobyłka, Poland), equipped with a reflux condenser, which was immersed into an ultrasonic bath (45 kHz, 350 W). An aqueous solution of inorganic base (30.00 mmol), a phase-transfer catalyst (0.15 mmol), CHP (1.50 mmol), and 1-bromobutane (1.50 mmol) were added, in this order, to the flask. The temperature was controlled and kept constant throughout the process (with a precision ±1 °C). After 1.5 h, the reaction mixture was cooled, and a sample was taken from the organic layer.

Analysis: Samples were analyzed by liquid chromatography UPLC (Waters Corp., Milford, MA, USA). The mobile phase was a mixture of acetonitrile:water (80:20 *v*/*v*), and the flow rate was 0.25 cm^3^/min. All peroxide yields were determined on the basis of UPLC analysis and calculated from the calibration curves of peroxide standards.

Catalyst recycling: Studies on the possibility of reusing the catalyst were carried out on a twice-larger scale, according to the procedure described above. After completion of the reaction, the mixture was cooled, and the catalyst phase was separated from the other two phases: organic and inorganic. The separated catalyst was used in the next process.

Product isolation: The organic phase was concentrated on a rotary evaporator (Heidolph Instruments GmbH & CO. KG, Schwabach, Germany). Purification of the product was carried out by column chromatography. Toluene was used as a mobile phase. ^1^H, ^13^ C NMR chemical shifts of dialkyl peroxides are available in the Supplementary Materials file. 

## 4. Conclusions

The combination of tri-liquid PTC system and ultrasound allowed for the development of an effective method of dialkyl peroxides synthesis. On the one hand, the formation of a third liquid phase by the catalyst simplifies the procedure for its separation and allows its use several times in subsequent processes. This, in turn, affects the reduction of investment and operating costs of the process. On the other hand, the use of ultrasound offers the intensification of the process, significantly reduces the reaction time, and allows peroxides to be obtained with high yields. In addition, PEGs used as catalysts are cheap, readily available, nontoxic, and easily biodegradable compounds. All this proves that the developed green approach has significantly practical appeal.

## Figures and Tables

**Table 1:** The effect of magnetic stirring or ultrasound irradiation. Reaction conditions: 1-bromobutane 0.21 g, 1.50 mmol; CHP 0.23 g, 1.50 mmol; PEG MM 2000 0.30 g, 0.15 mmol; 50% KOH 1.68 g, 30.00 mmol; cyclohexane 3 cm^3^; temperature 50 °C; selectivity 100%.

Technique	Time (h)	Peroxide Yield (%)
Magnetic stirring, 500 rpm	5.0	97
Ultrasound irradiation	1.5	99

**Table 2:** The effect of end groups and chain length of polyethylene glycols. Reaction conditions: 1-bromobutane 0.21 g, 1.50 mmol; CHP 0.23 g, 1.50 mmol; catalyst 0.15 mmol; 50% KOH 1.68 g, 30.00 mmol; cyclohexane 3 cm^3^; temperature 50 °C; reaction time 90 min; ultrasonic bath 45 kHz, 350 W; selectivity 100%.

Entry	Catalyst	Amount of Catalyst (% mol)	n~:^1^	Peroxide Yield (%)
1	PEG 200	10	4	18
2	PEG 600	10	13	70
3	PEG 1000	10	22	81
4	PEG MM 350	10	7	71
5	PEG MM 550	10	12	74
6	PEG MM 1100	10	24	85
7	PEG MM 2000	10	44	99
8	PEG MM 2000	5	44	78
9	PEG MM 2000	1	44	77
10	PEG DM 500	10	10	57
11	PEG DM 1000	10	21	92

^1^ n—number of ethoxylate group in PEGs and their derivatives X(CH_2_CH_2_O)_n_Y, where X is –OH or –OCH_3_, and Y is –H or –CH_3_.

**Table 3:** The effect of end groups and chain length of polyethylene glycols. Reaction conditions: 1-bromobutane 0.21 g, 1.50 mmol; CHP 0.23 g, 1.50 mmol; PEG MM 2000 0.30 g, 0.15 mmol; cyclohexane 3 cm^3^; temperature 50 °C; reaction time 90 min; ultrasonic bath 45 kHz, 350 W; selectivity 100%.

Entry	Base	Concentration of Base Solution	Molar Ratio KOH:CHP	Peroxide Yield (%)
1 2 3 4 5 6	NaOH KOH KOH KOH KOH KOH	50 50 40 30 50 50	20 20 20 20 10 5	67 99 71 60 83 81

**Table 4:** The effect of a kind of organic solvent. Reaction conditions: 1-bromobutane 0.21 g, 1.50 mmol; CHP 0.23 g, 1.50 mmol; PEG MM 2000 0.30 g, 0.15 mmol; 50% KOH 1.68 g; organic solvent 3 cm^3^; temperature 50 °C; reaction time 90 min; ultrasonic bath 45 kHz, 350 W; selectivity 100%.

Entry	Solvent	Peroxide Yield (%)
1	cyclohexane	99
2	toluene	35

**Table 5:** The effect of a kind of organic solvent. 1 alkyl bromide, 1.50 mmol; CHP 0.23 g, 1.50 mmol; PEG MM 2000 0.30 g, 0.15 mmol; 50% KOH 1.68 g; cyclohexane 3 cm^3^; temperature 50 °C; reaction time 90 min; ultrasonic bath 45 kHz, 350 W; selectivity 100%.

Alkyl Bromide	Peroxide Yield (%)
n-bromopropane 2-bromopropane n-bromobutane 2-bromobutane n-bromopentane n-bromohexane	96 (fresh catalyst) 93 (1th cycle) 89 (2nd cycle) 87 (3rd cycle) 75 99 (fresh catalyst) 95 (1th cycle) 91 (2nd cycle) 89 (3rd cycle) 70 88 85

## Supplementary Materials

The following are available online. ^1^H, ^13^ C NMR chemical shifts of dialkyl peroxides.
