# Supplementary material for: Ultrasound-Assisted Green Synthesis of Dialkyl Peroxides under Phase-Transfer Catalysis Conditions
Source: Molecules. 2019 Dec 28;25(1):118. doi: 10.3390/molecules25010118 (PMC6982749; doi:10.3390/molecules25010118)
Supplement: Supplementary file 1 [file molecules-25-00118-s001.pdf]

Supplementary materials

Ultrasound-assisted green synthesis of dialkyl peroxides under phase-transfer catalysis conditions

Daniel Kopeć, Stefan Baj and Agnieszka Siewniak*

Silesian University of Technology, Department of Chemical Organic Technology and Petrochemistry, Krzywoustego 4, 44-100 Gliwice, Poland.

Correspondence to: A. Siewniak, Silesian University of Technology, Department of Chemical Organic Technology and Petrochemistry, Krzywoustego 4, 44-100 Gliwice, Poland. Email: agnieszka.siewniak@polsl.pl

(1-phenyl-1-methyletyl)-1-propyl peroxide (CAS registry number: 28009-66-1)

δ_H (300 MHz, $CDCl_3$): 7.36 (5H, m), 3.68 (2H, t, J = 6.8), 1.54 (2H, m), 0.96 (3H, t, J = 7.0).

δ_C (75 MHz, $CDCl_3$): 145.7; 128.3, 127.8, 126.8, 82.6, 77.1, 26.6, 21.2, 9.3.

(1-phenyl-1-methyletyl)-2-propyl peroxide (CAS registry number: 28009-68-9)

δ_H (300 MHz, $CDCl_3$): 7.36 (5H, m), 4.14 (1H, sept, J = 6.0), 1.59 (6H, s), 1.14 (6H, d, J = 6.6).

δ_C (75 MHz, $CDCl_3$): 145.6; 127.9; 126.9; 125.5; 82.3; 75.7; 26.6; 20.4.

1-butyl-(1-phenyl-1-methyletyl) peroxide (CAS registry number: 1080-45-1)

δ_H (300 MHz, $CDCl_3$): 7.41 (5H, m), 3.92 (2H, t, J = 6.8), 1.65 (6H, s), 1.58 (2H, m), 1.35 (2H, sec., J = 7.5), 0.92 (3H, t, J = 7.3).

δ_C (75 MHz, $CDCl_3$): 145.5; 128.0, 127.0, 125.5, 82.6, 74.6, 29.9, 26.5, 19.3, 13.8.

2-butyl-(1-phenyl-1-methyletyl) peroxide (CAS registry number: 61808-95-5)

δ_H (300 MHz, $CDCl_3$): 7.36 (5H, m), 3.93 (1H, sextet, J = 6.6), 1.60 (6H, s), 1.38 (2H, m), 1.15 (3H, d, J = 6.3); 0.81 (3H, t, J = 7,2).

δ_C (75 MHz, $CDCl_3$): 146.0; 128.2, 127.2, 125.8, 82.6, 75.5, 27.6, 26.8, 18.4, 10.1.

(1-phenyl-1-methyletyl)-1-pentyl peroxide (CAS registry number: 30312-21-1)

δ_H (300 MHz, $CDCl_3$): 7.39 (5H, m), 3.94 (2H, t, J = 6.6), 1.62 (6H, s), 1.56 (2H, m), 1.27 (4H, m), 0.88 (3H, t, J = 6.8).

δ_C (75 MHz, $CDCl_3$): 145.5; 128.0, 127.0, 125.5, 82.6, 74.9, 28.2, 27.5, 26.5, 22.4, 13.9.

1-hexyl-(1-phenyl-1-methyletyl) peroxide (CAS registry number: 157793-71-0)

δ_H (300 MHz, $CDCl_3$): 7.40 (5H, m), 3.94 (2H, t, J = 6.6), 1.62 (6H, s), 1.56 (2H, m), 1.28 (6H, m), 0.89 (3H, t, J = 7.0).

δ_C (75 MHz, $CDCl_3$): 145.5; 128.0, 127.0, 125.59, 82.6, 74.9, 31.6, 27.8, 26.5, 25.8, 22.5, 14.0.
